# Systematic review and meta-analysis of mortality of patients infected with carbapenem-resistant *Klebsiella pneumoniae*

**DOI:** 10.1186/s12941-017-0191-3

**Published:** 2017-03-29

**Authors:** Liangfei Xu, Xiaoxi Sun, Xiaoling Ma

**Affiliations:** Department of Laboratory Medicine, Anhui Provincial Hospital, Anhui Medical University, Hefei, 230001 Anhui China

**Keywords:** CRKP, Carbapenem-resistant, *K. pneumoniae*, Mortality

## Abstract

**Purpose:**

Carbapenem resistant *K. pneumoniae* (CRKP) has aroused widespread attention owing to its very limited therapeutic options, and this strain has increased rapidly in recent years. Although it is accepted that drug resistance is associated with increased mortality in general, but some other studies found no such relationship. To estimate mortality of patients infected with CRKP in general and analyze factors for mortality of this infection, thus, we conducted this systematic review and meta-analysis.

**Methods:**

A systematic literature review of relevant studies published until December 2015 was conducted. We selected and assessed articles reporting mortality of patients infected with CRKP.

**Results:**

Pooled mortality was 42.14% among 2462 patients infected with CRKP versus 21.16% in those infected with carbapenem-susceptible *K. pneumoniae* (CSKP). The mortality of patients with bloodstream infection (BSI) or urinary tract infection was 54.30 and 13.52%, respectively, and 48.9 and 43.13% in patients admitted to the intensive care unit (ICU) or who underwent solid organ transplantation (SOT). Mortality was 47.66% in patients infected with *K. pneumoniae* carbapenemase-producing *K. pneumoniae* and 46.71% in those infected with VIM-producing *K. pneumoniae.* Geographically, mortality reported in studies from North America, South America, Europe, and Asia was 33.24, 46.71, 50.06, and 44.82%, respectively.

**Conclusions:**

Our study suggests that patients infected with CRKP have higher mortality than those infected with CSKP, especially in association with BSI, ICU admission, or SOT. We also considered that patients’ survival has a close relationship with their physical condition. Our results imply that attention should be paid to CRKP infection, and that strict infection control measures and new antibiotics are required to protect against CRKP infection.

## Background

It is well known that *Klebsiella pneumoniae* is ubiquitous in nature, one of the most relevant opportunistic pathogens, and causes various human infections such as bloodstream infection (BSI), urinary tract infection (UTI), surgical-site infection, and pneumonia [1–3]. Resistance can develop in *K. pneumoniae* isolates, notably producing extended-spectrum β-lactamases (ESBLs). ESBL-producing strains of *K. pneumoniae* are currently found throughout the world and have caused numerous outbreaks of infection [4, 5]. Carbapenems represent the first-line therapy for severe infection by ESBL-producing *K. pneumoniae* [6]. However, since Yigit et al. [7, 8] reported the first *K. pneumoniae* carbapenem (KPC)-producing *K. pneumoniae* isolate in North Carolina in 1996, carbapenem-resistant strains have increased rapidly, rising from 1.6 to 10.4% associated with central line blood-stream infections between 2001 and 2011 in the United States, and have aroused widespread attention, presenting a challenge because the antimicrobial treatment options remain very restricted [7, 9].

Carbapenem-resistant *K. pneumoniae* (CRKP) deactivates the carbapenems through two main mechanisms: (1) acquisition of carbapenemase genes that encode for enzymes capable of hydrolyzing carbapenems—the three most important carbapenemase types being KPC-type enzymes, metallo-β-lactamases (VIM, IMP, NDM), and OXA-48 type enzymes; and (2) reduction in the accumulation of antibiotics by a quantitative and/or qualitative deficiency of porin expression in combination with overexpression of β-lactamases that possess weak affinity for carbapenems [10].

Most researchers reported higher mortality rates among persons infected with CRKP isolates [11–30] while others reported contrary results [31, 32]. In recent years, many studies from single medical centers or individual countries have reported mortality rates in patients infected with CRKP, but until now there has been no systematic review focusing on mortality resulting from carbapenem-resistant infections in general. Although in a recent meta-analysis Falagas et al. [33] reported a higher all-cause mortality among patients infected with carbapenem-resistant Enterobacteriaceae than in those with carbapenem-susceptible infections, but their research included only nine studies. Considering this scenario, we conducted a systematic review and meta-analysis to estimate the mortality of patients infected with CRKP, and analyzed mortality resulting from multiple infection types and patients conditions.

## Methods

### Search strategy

Two independent examiners (LF.X. and XX.S.) searched entries in the PubMed and EMBASE databases from their inception until December 22, 2015 to identify potentially relevant studies. The search terms included “*Klebsiella pneumoniae*” AND resistance AND (“carbapenem” OR “imipenem” OR “meropenem” OR “ertapenem”). The language was restricted to English.

### Inclusion and exclusion criteria

Studies were considered in accordance with inclusion criteria if articles reported mortality of patients infected with CRKP. Research that focused on children, did not differentiate mortality between infection and colonization, did not define the strains that were carbapenem resistant, and did not present the exact death toll were excluded. In this analysis, carbapenem resistance was defined as resistance to carbapenems such as imipenem, meropenem, and ertapenem, irrespective of susceptibility to other antibiotics.

### Assessment of study quality

The articles were assessed for quality of the cohort or case–control studies included in the systematic analysis according to the Newcastle-Ottawa scale (NOS) score [34], ranging from 0 to 9. Studies with a NOS score of 5 or greater were included in this analysis.

### Data extraction

Two independent investigators (LF.X. and XX.S.) extracted information from eligible articles. Divergences were solved by discussion and consultation of the relevant literature. The information extracted from original publications included title, first author, year of publication and experiment, type of study, sample size, characteristics of the study population (mean age, sex, type of infection, mean severity of underlying disease), and crude mortality rates in patients infected with CRKP and carbapenem-susceptible *K. pneumoniae* (CSKP). If articles reported mortality from both infection and colonization, we extracted information only regarding infections.

### Statistical analysis

We calculated the pooled odds ratio (OR) and 95% confidence interval (CI) by comparing crude mortality in patients with CRKP with that in patients with CSKP. Between-study heterogeneity was assessed by the χ^2^ test (*p* < 0.10 was selected to indicate the presence of heterogeneity, in which case a random-effects model was adopted; otherwise a fixed-effects model was applied) and *I* ^2^ test (to assess the degree of heterogeneity) [35, 36]. We then calculated pooled rates of mortality in patients infected with CRKP, and stratified analyses with respect to geographic location, infection types, carbapenemase types, and patients conditions performed. Freeman–Tukey arcsine transformations were used to stabilize the variances, and after the meta-analysis we transformed the summary estimates and the CI boundaries back to proportions using the sine function [37]. We used Stata version 12.0 software for all statistical calculations.

## Results

### Results of the systematic literature search

**Fig. 1:**
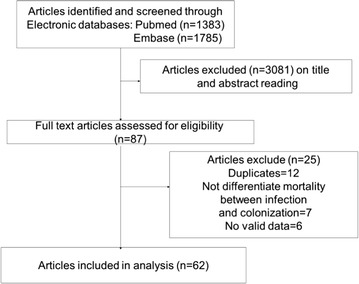
Flow diagram of included studies

We identified and screened 3168 articles. After exclusion by title and abstract, the remaining 87 articles were subjected to full-text assessment for eligibility. Among these articles, 12 were duplicates, seven did not differentiate between infection- and colonization-related mortality, and six did not report valid data. Ultimately, 62 studies were analyzed based on the inclusion and exclusion criteria (Fig. 1).

The basic characteristics of these 62 studies are summarized in Table 1 [11–32, 38–77]. These articles were published from 1999 to 2015 and the sample size varied across studies, ranging from 7 to 1022. The total number of patients in this systematic review was 4701, of whom 2462 had CRKP infection and the remainder CSKP infection. Among these patients, the reported death was 1018 among the CRKP patients and 398 among the CSKP patients. In the pooled analysis, the overall mortality was 42.14% (95% CI 37.06–47.31) in patients infected with CRKP and 21.16% (95% CI 16.07–26.79) in CSKP patients (Table 2).

**Table 1:** Characteristics of the eligible studies

Author, year	Study type	Region/study year	Resistance	CRKP mortality (%)	CSKP mortality (%)	P value	Carbapenemases	Infection type	ICU	SOT
Vardakas (2015) [11]	Retrospective cohort study	Greece 2006.1–2009.10	CLSI 2010	58/80 (72.5)	14/24 (58.3)	0.19	NA	BSI:44/65	80	0
Brizendine (2015) [16]	Retrospective cohort study	USA 2011.12–2013.10	CLSI 2012	16/157 (10.2)	NA	NA	NA	UTI:16/157	0	0
Pouch (2015) [12]	Nested case–control study	USA 2007.1–2010.12	CLSI 2009	6/20 (30)	8/80 (10)	0.03	NA	UTI:6/20	0	20
Ny (2015) [13]	Retrospective cohort study	USA 2011.1–2013.12	NA	7/48 (14.6)	5/48 (10.4)	0.76	NA	UTI:2/27	0	0
Girmenia (2015) [39]	Retrospective cohort study	Italy 2010.1–2013.7	NA	65/112 (58.1)	NA	NA	NA	Any infection:65/112	0	112
Hoxha (2015) [14]	Prospective matched cohort study	Italy 2012.11–2013.7	Eucast Guideline	30/49 (61)	10/49 (20)	NA	NA	Any infection:30/49	0	0
Cubero (2015) [15]	Retrospective cohort study	Spain 2010.10–2012.12	EUCAST 2015	8/20 (40)	1/9 (11.1)	NA	NA	Any infection:8/20	0	0
Chang (2015) [40]	Retrospective study	Taiwan 2012.1–2012.12	CLSI 2012	21/41 (51.2)	NA	NA	KPC:6/8	Any infection:21/41	41	0
Chen (2015) [68]	Retrospective study	Taiwan 2014.4–10	NA	12/41 (29.3)	NA	NA	NA	Any infection:12/41	0	0
Madrigal (2015) [66]	Retrospective study	Spain 2014.5–9	NA	2/5 (40)	NA	NA	NA	Any infection:2/5	0	0
Bias (2015) [70]	Retrospective, observational cohort study	USA –2014.8	NA	5/30 (16.7)	NA	NA	NA	Any infection:5/30	0	30
Katsiari (2015) [67]	Prospective, observational study	Greece 2010.4–2012.3	CLSI 2012	14/32 (43.8)	NA	NA	KPC:11/28VIM:3/5	BSI:9/16	32	0
Maristela Freire (2015) [69]	Retrospective cohort study	Brazil 2009.1–2013.12	CLSI 2012	13/31 (41.9)	NA	NA	KPC:13/31	BSI:7/11 UTI:1/10	0	31
Brizendine (2015) [16]	Retrospective cohort study	USA 2006–2012	NA	4/22 (18)	1/64 (1.5)	NA	NA	UTI:4/22	0	22
Sarah Welch (2015) [65]	Retrospective cohort study	USA	NA	19/51 (37.3)	NA	NA	NA	Pneumonia:19/51	0	0
van Duin (2014) [16]	Prospective, multi-center, observational study	USA 2011.12–2013.3	CLSI	26/114 (22.8)	NA	NA	NA	BSI:5/26	0	0
Simkins (2014) [17]	Retrospective case–control study	USA 2006.1–2010.12	NA	6/13 (46.2)	3/39 (7.7)	0.005	NA	Any infection:6/13	0	13
Viviana Gómez Rueda (2014) [18]	Case–case–control study	Colombia 2008.1–2011.1	CLSI	31/61 (50.8)	20/61 (32.8)	NA	NA	Any infection:31/61	0	0
Christoph Lübbert (2014) [71]	Retrospective study	Germany 2010.9–2011.9	NA	7/8 (87.5)	NA	NA	KPC:7/8	Any infection:7/8	0	8
Qureshi (2014) [42]	Retrospective cohort study	USA 2009.1–2012.10	NA	0/21 (0.00)	NA	NA	NA	UTI:0/21	0	0
Mouloudi (2014) [43]	Retrospective cohort study	Greece 2008.1–2011.12	EUCAST 2012	14/17 (82.4)	NA	NA	NA	BSI:14/17	17	17
Bulent Aydinl (2014) [72]	Retrospective analysis	Turkey 2012.1–2013.11	NA	2/5 (40)	NA	NA	NA	Any infection:2/5	0	5
Gallagher (2014) [44]	Retrospective case–case–control study	USA 2005.6–2010.10	CLSI 2009	19/43 (44.2)	NA	NA	NA	BSI:19/43	0	0
Graziella Hanna Pereira (2013) [47]	Retrospective cohort study	Brazil 2008.10–2010.10	CLSI 2010	16/33 (48)	NA	NA	NA	BSI:9/11 UTI:3/21 Pneumonia:3/7	0	0
Orsi (2013) [19]	Case–case–control study	Italy 2008.7–2011.6	EUCAST	25/65 (38.5)	12/43 (27.9)	NA	KPC:14/36	Any infection:25/65	0	0
Kontopidou (2013) [48]	Retrospective cohort study	Greece 2009.9–2010.6	CLSI 2010	29/127 (22.8)	NA	NA	NA	Any infection:29/127	127	0
Hussein (2013) [20]	Retrospective case control study	Israel 2006.1–2008.12	CLSI 2006	45/103 (43.7)	62/214 (29)	NA	NA	BSI:45/103	0	0
Luci Correa (2013) [22]	Matched case–control study	Brazil 2006.1–2008.8	CLSI 2009	10/20 (50)	11/40 (27.5)	0.085	NA	Any infection:10/20	0	0
Clancy (2013) [49]	Single-center, retrospective study	USA 2008.8–2011.7	CLSI 2012	3/17 (17.6)	NA	NA	NA	BSI:3/17	0	17
Cober (2013) [21]	Retrospective cohort study	USA 2006–2009	NA	8/19 (42.1)	7/46	0.005	NA	BSI:8/19	0	19
Grossi (2013) [73]	Retrospective cohort study	Italy 2009.1–2012.10	NA	11/36 (30.6)	NA	NA	NA	Any infection:11/36	0	36
Cicora (2013) [50]	Observational, retrospective study	Argentina 2011.4–2012.6	CLSI 2010	2/6 (33.3)	NA	NA	KPC:2/6	UTI:2/6	0	6
Paola Di Carlo (2013) [46]	Prospective case series study	Italy 2011,8–2012.8	EUCAST	12/30 (40)	NA	NA	KPC:12/30	Any infection:12/30	30	0
Fligou (2013) [88]	Retrospective cohort study	Greece	CLSI	21/48 (43.8)	NA	NA	KPC:21/48	BSI:21/48	48	0
Rose (2012) [74]	Retrospective, cohort study	USA 2006–2011	NA	20/44 (45.5)	NA	NA	NA	BSI:20/44	0	0
Sanchez-Romero (2012) [51]	Retrospective cohort study	Spain 2009.1–2009.12	CLSI 2011	13/28 (46.4)	NA	NA	VIM:13/28	Any infection:13/28	28	0
Liu (2012) [23]	Matched case–control study	Taiwan 2007.1–2009.12	CLSI 2009	15/25 (60)	20/50	0.102	NA	BSI:15/25	0	0
Kalpoe (2012) [52]	Retrospective cohort study	USA 2005.1–2006.10	NA	10/14 (71.4)	NA	NA	NA	Any infection:10/14	0	14
Borer (2012) [53]	Retrospective case control study	Israel 2007.5–2010.1	CLSI 2006	13/42 (31)	NA	NA	NA	Any infection:13/42	0	0
Bergamasco (2012) [54]	Retrospective cohort study	Brazil 2009.7–2010.2	CLSI 2009	5/12 (41.7)	NA	NA	KPC:2/12	Any infection:5/12	0	12
Ben-David (2012) [24]	Retrospective cohort study	Israel 2006.1–2006.12	CLSI 2006	29/42 (69.1)	45/150 (30)	<0.001	NA	BSI:29/42	0	0
Balkhy (2012) [55]	Retrospective/prospective surveillance study	Saudi Arabia 2009.9–2010.8	CLSI 2009	8/20 (40)	NA	NA	NA	Any infection:8/20	0	0
Jason Gallagher (2011) [75]	A retrospective, cohort study	USA 2006–2011	NA	24/44 (54.5)	NA	NA	NA	BSI:24/44	0	0
Pereira (2011) [56]	Retrospective cohort study	Brazil 2008.10–2010.8	CLSI 2010	9/22 (40.9)	NA	NA	NA	Any infection:9/22	0	0
Orsi (2011) [25]	Retrospective case control study	Italy 2008.7–2009.12	EUCAST	11/28 (39.3)	12/43	NA	NA	Any infection:11/28	0	0
Neuner (2011) [57]	Retrospective cohort study	USA 2007.1–2009.5	CLSI 2009	35/60 (58.3)	NA	NA	NA	BSI:35/60	0	0
Diana Gaviria (2011) [31]	Retrospective matched case–control study	USA 2009.4–2011.12	CLSI	1/19 (5.3)	3/38 (7.9)	NA	NA	Any infection:1/19	0	0
Cuzon (2011) [59]	Retrospective cohort study	France 2010.4–2010.6	CLSI 2010	5/7 (71.4)	NA	NA	NA	Any infection:5/7	0	0
Elisa Maria Beirão (2011) [58]	Retrospective cohort study	Brazil 2008.1–2008.12	CLSI 2009	3/6 (50)	NA	NA	KPC:3/6	Any infection:3/6	0	0
Nguyen (2010) [60]	Retrospective cohort study	USA 2004.1–2008.9	CLSI	29/48 (60.4)	NA	NA	NA	BSI:29/48	0	0
Vardakas (2010) [76]	Retrospective cohort study	Greece 2006.1–2009.9	NA	42/56 (75)	NA	NA	NA	Any infection:42/56	56	0
Mouloudi (2010) [26]	Retrospective nested case–control study	Greece 2007.1–2008.12	CLSI 2007	25/37 (67.6)	9/22 (40.9)	0.03	KPC: 15/19 VIM:10/18	BSI:25/37	0	0
Gregory (2010) [61]	Retrospective case–control study	Puerto Rico 2008.2–2008.9	CLSI 2009	7/19 (36.8)	NA	NA	NA	Any infection:7/19	0	0
Balandin Moreno (2010) [77]	Retrospective cohort study	Spain 2009.7–2010.4	NA	2/8 (25)	NA	NA	VIM:2/8	Any infection:2/8	8	0
Gasink (2009) [27]	Case–control study	USA 2006.10–2008.4	NA	18/56 (32.1)	85/863 (9.8)	NA	KPC:18/56	Any infection:18/56	0	0
Daikos (2009) [28]	Prospective observational study	Greece 2005.2–2006.3	CLSI 2004	6/14 (42.9)	25/148 (16.9)	NA	VIM:6/14	BSI:6/14	0	0
Borer (2009) [62]	Matched retrospective, historical cohort study	Israel 2005.10–2008.10	CLSI 2006	30/64 (46.9)	NA	NA	NA	BSI:23/32	0	0
Schwaber (2008) [29]	Retrospective cohort study	Israel 2003–2006	CLSI 2005	21/48 (43.8)	7/56 (12.5)	NA	NA	Any infection:21/48	0	0
Patel (2008) [30]	Retrospective matched case–control	USA 2004.7–2006.6	CLSI 2006	48/99 (48.5)	20/99 (20.2)	<0.001	NA	Any infection:48/99	0	0
Falagas (2007) [32]	Retrospective matched case–control study	Greece 2000.10–2006.5	NA	16/53 (30.2)	18/53 (34)	0.83	NA	Any infection:16/53	0	0
Woodford (2004) [63]	Retrospective cohort study	USA 2000.4–2001.4	CLSI	8/14 (57.1)	NA	NA	KPC:8/14	Any infection:8/14	14	0
Muhammad Ahmad. (1999) [64]	Retrospective cohort study	USA 1994.12–1995.11	CLSI 1994	6/8 (75)	NA	NA	NA	Any infection:6/8	8	0

*CLSI* Clinical and Laboratory Standards Institute, *CRKP* carbapenem-resistant *K. pneumoniae*, *CSKP* carbapenem-susceptible *K. pneumonia*, *BSI* bloodstream infection, *UTI* urinary tract infection

**Table 2:** Mortality of patients based on patient condition, carbapenemases type, study region

Subgroup	Number of studies	Sample size	Mortality Rate %(95% CI)	Statistical model
Pooled mortality	P < 0.001			
CRKP	62	2462	42.14 (37.06–47.31)	Random
CSKP	22	2239	21.12 (16.07–26.79)	Random
Patient conditions	P < 0.001			
Bloodstream infections	20	722	54.30 (47.51–61.02)	Random
Urinary tract infections	8	284	13.52 (7.50–20.92)	Random
Intensive care unit	12	479	53.90 (39.44–68.00)	Random
Solid organ transplantation	15	362	43.13 (32.40–54.16)	Random
Carbapenemases type	P = 0.645			
KPC-producing *Klebsiella pneumoniae*	13	302	47.66 (38.61–49.51)	Random
VIM-producing *Klebsiella pneumoniae*	5	73	46.71 (35.81–57.73)	Random
Region	P = 0.062			
North America	23	980	33.24 (25.08–42.00)	Random
South America	8	191	46.71 (39.83–53.66)	Fixed
Europe	21	860	50.06 (41.45–58.62)	Random
Asia	10	431	44.82 (37.83–51.91)	Random

*CRKP* Carbapenem-resistant *K. pneumoniae*, *CSKP* carbapenem-susceptible *K. pneumonia*

### Comparison of mortality in CRKP and CSKP patients

**Fig. 2:**
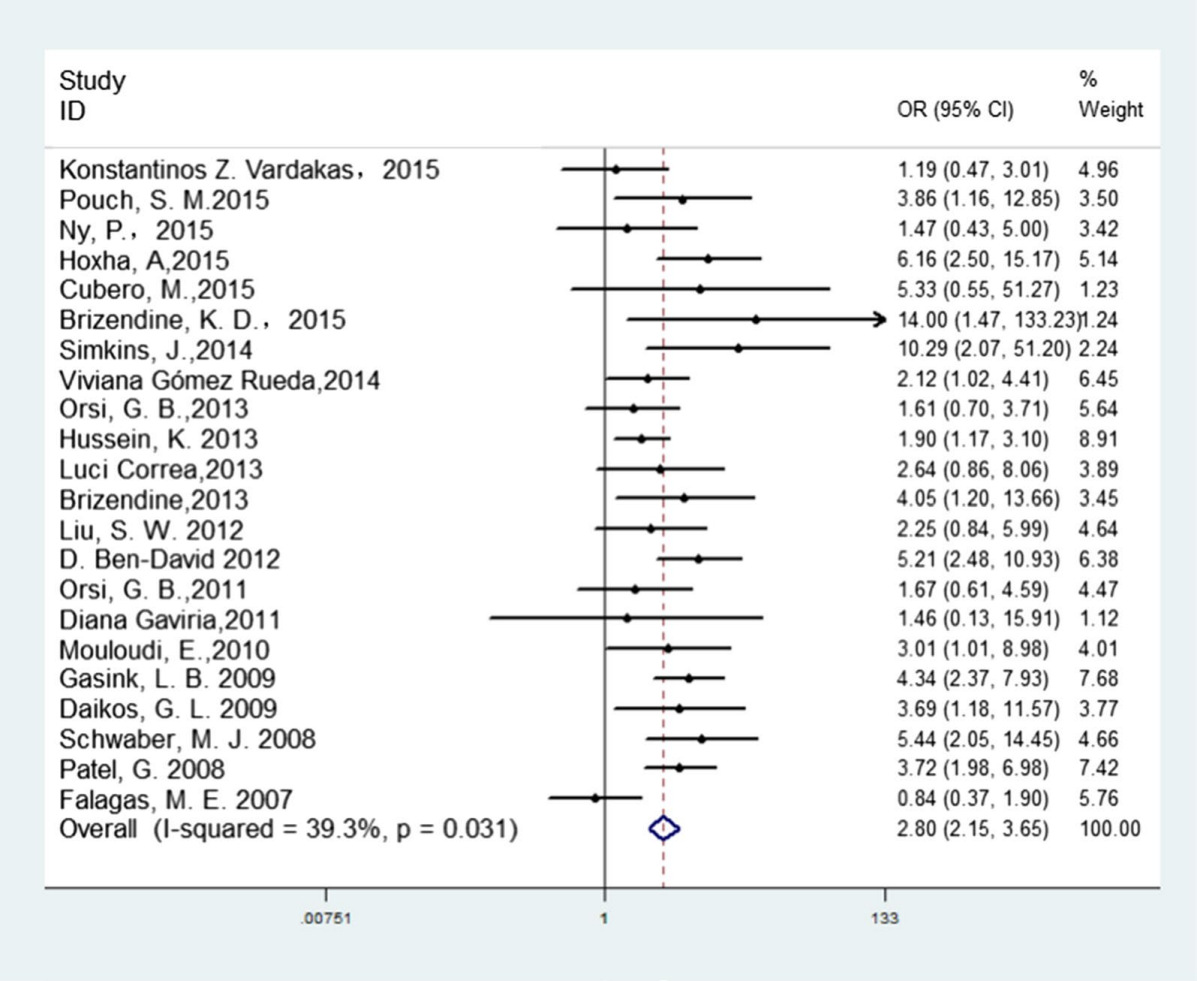
Crude odds ratio (OR) for the association between carbapenem resistance and mortality of patients with *K. pneumoniae* infection

Among the included articles, 22 compared mortality between patients infected with CRKP and CSKP. The summary estimate of these studies from the random-effects model suggested that patients with CRKP had a significantly higher mortality than those with CSKP in the univariate analysis (pooled crude OR 2.80; 95% CI 2.15–3.65) with a moderate heterogeneity *I* ^2^ of 33.9% (*p* = 0.031) (Fig. 2).

### Mortality in multiple patient conditions

As shown in Table 2, 722 patients had BSI and 284 had UTI, 479 were in an intensive care unit (ICU), and 362 underwent solid organ transplantation (SOT). In the pooled analysis, the mortality was 54.30% (95% CI 47.51–61.02), 13.52% (95% CI 7.50–20.92), 48.9% (95% CI 44.47–53.46), and 43.13% (95% CI 32.40–54.16) in BSI, UTI, ICU-admission, and SOT patients, respectively.

### Mortality in multiple carbapenemase types

In this subgroup analysis, we mainly analyzed the mortality of patients infected with KPC-producing *K. pneumoniae* and VIM-producing *K. pneumoniae.* In the articles included, 302 patients were infected with KPC-producing *K. pneumoniae* and 73 were infected with VIM-producing *K. pneumoniae*. The mortality among these two types of carbapenemases was 47.66% (95% CI 38.61–56.79) and 46.71% (95% CI 35.81–57.73), respectively (Table 2).

### Mortality in different geographic locations

Twenty-three studies were carried out in North America, eight in South America, twenty-one in Europe, and ten in Asia. The rate of mortality was 33.24% (95% CI 25.08–42.00) of 980 patients in North America, 46.71% (95% CI 39.83–53.66) of 191 in South America, 50.06% (95% CI 41.45–58.62) of 860 in Europe, and 44.82% (95% CI 37.83–51.91) of 431 in Asia (Table 2).

## Discussion

ESBL-producing *K. pneumoniae* as an opportunistic pathogen is becoming more challenging to treat because of the emergence of carbapenem resistance, and has a significant influence on patient mortality. The primary result of this analysis was the pooled crude mortality of 42.14% among patients with CRKP, which is intimately connected with patients’ health and physical status.

Although it is accepted that drug resistance is associated with increased mortality because patients tend to receive inappropriate empiric therapy in general [4, 78], other studies have found no such relationship. Bhavnani et al. [79] reported that clinical success was similar between patients with ESBL and those with non-ESBL-producing *K. pneumoniae*, and ESBL production alone did not appear to be an independent risk factor for treatment failure. Kim et al. [80] also found that ESBL production was not significantly associated with death. In addition, García-Sureda et al. [81] reported that CRKP isolates are less virulent and fit than CSKP isolates in an antibiotic-free environment. We conducted this systematic review and meta-analysis to estimate the mortality of patients infected with CRKP in general and to study the factors related to mortality resulting from this infection. We found that patients infected with CRKP had significantly higher mortality in comparison with CSKP (crude OR 2.80). To identify risk factors associated with the higher mortality of CRKP infections, we conducted a stratified analysis of patient condition, carbapenemase types, and study location.

Based on multiple patient conditions, our analysis confirmed that patients with CRKP in association with BSI, ICU admission, or SOT have a higher mortality than the pooled mortality, although UTI patients have a lower mortality than the pooled overall mortality, even lower than that of CSKP patients. From this result, we assumed that patient survival has a close relationship with patients’ underlying illness and comorbidities. Mouloudi et al. [26] reported that BSI, ICU admission, and recent receipt of a SOT were associated with ICU and in-hospital mortality in patients infected with CRKP. In addition, patients who had undergone organ transplantation or ICU admission were always subjected to surgical procedures, prolonged ICU stay, preexisting immunosuppression, and the use of invasive devices, which contributed to patients’ poor physical condition and resultant higher mortality. In contrast, Daikos et al. suggested that UTI is a relatively mild infection that has only a slight influence on the general condition of patients, and carries a low mortality in general [25]. It has been shown that factors such as underlying illness and comorbidities have a more important influence on mortality than appropriate empiric treatment with multidrug-resistant Gram-negative bacteria [82]. Although the underlying patient’s condition is important for the outcome of such patients, meanwhile a timely effective treatment can also help to improve the survival rate. Patients in a poor state of health with CRKP were subjected to pathogens longer compared to CSKP infection due to lack of an effective therapy, ultimately, led to a higher mortality.

In the present analysis, patients infected with KPC-producing *K. pneumoniae* have a higher mortality than pooled overall mortality (47.66 vs 42.14%). This result may contribute to KPC-producing *K. pneumoniae* having stronger invasiveness, and the KPC-encoding *blaKPC* always carry other drug-resistant genes, leading to a pronounced drug resistant [83]. Previous studies have demonstrated *K. pneumoniae*-encoding *blaKPC* to be an independent risk factor in patient mortality [26, 27]. In addition, KPC-producing *K. pneumoniae* is considered a successful pathogen because of its ability to persist and spread, causing nosocomial outbreaks. Bratu et al. [84] reported that KPC-producing *K. pneumoniae* isolates are resistant to not only all β-lactam antimicrobials but also frequently other classes of antimicrobials, such as aminoglycosides and fluoroquinolones. In this systematic review, the patients from North America have lower mortality in comparison with the other three locations. This phenomenon may be attributed to a higher level of medical care and different treatment methods in North America, such as combination antibiotics, treatment with polymyxins and tigecycline, and adjunctive procedures (e.g., catheter removal, drainage, or debridement). There is evidence that tigecycline and polymyxins have activity against many CRKP isolates in vitro, and there have been cases reported of successful treatment of CRKP infection with polymyxins and tigecycline [85–87]. Patel et al. [30] also reported that removal of the focus of infection (i.e., debridement) was independently associated with patient survival.

There are several limitations to this analysis. First, as the included studies reported only unadjusted data on mortality, we analyzed only crude mortality among patients with CRKP. Second, most studies may have lacked power in differentiating death caused by CRKP from any other factors, and it is difficult to draw definitive conclusions from current evidence because of the residual confounding factors and small sample sizes in many studies. Third, some studies included in our meta-analysis did not define a cutoff value to judge the susceptibility of *K. pneumoniae* to carbapenems, and when defined the cutoff value varied among studies owing to different reference criteria. Thus, there exists the potential for heterogeneity. Fourth, most studies were retrospective in nature and thus susceptible to selection bias. Last, we selected only English-language articles, thus limiting the scope of our analysis.

## Conclusions

Our study suggests that patients infected with CRKP have a higher mortality than those infected with CSKP, especially patients with BSI, ICU admission, or SOT intervention. We suggest that the survival of patients has a close relationship with their physical condition. Thus, our results imply that attention should be paid to CRKP infection in patients in a poor state of health, and that strict infection control measures and new antibiotics are required to protect against CRKP infection.

## Abbreviations

CRKP: carbapenem-resistant *Klebsiella pneumoniae*
CSKP: carbapenem-suscepyible *Klebsiella pneumoniae*
BSI: bloodstream infection
UTI: urinary tract infection
ICU: intensive care unit
SOT: solid organ transplantation
